# No Evidence for Strong Recent Positive Selection Favoring the 7 Repeat Allele of VNTR in the *DRD4* Gene

**DOI:** 10.1371/journal.pone.0024410

**Published:** 2011-08-31

**Authors:** Izumi Naka, Nao Nishida, Jun Ohashi

**Affiliations:** 1 Doctoral Program in Biomedical Sciences, Graduate School of Comprehensive Human Sciences, University of Tsukuba, Ibaraki, Japan; 2 Department of Human Genetics, Graduate School of Medicine, The University of Tokyo, Bunkyo-ku, Tokyo, Japan; 3 Research Center for Hepatitis and Immunology, International Medical Center of Japan Konodai Hospital, Ichikawa, Chiba, Japan; Institut Pasteur, France

## Abstract

The human dopamine receptor D4 (*DRD4*) gene contains a 48-bp variable number of tandem repeat (VNTR) in exon 3, encoding the third intracellular loop of this dopamine receptor. The *DRD4* 7R allele, which seems to have a single origin, is commonly observed in various human populations and the nucleotide diversity of the *DRD4* 7R haplotype at the *DRD4* locus is reduced compared to the most common *DRD4* 4R haplotype. Based on these observations, previous studies have hypothesized that positive selection has acted on the *DRD4* 7R allele. However, the degrees of linkage disequilibrium (LD) of the *DRD4* 7R allele with single nucleotide polymorphisms (SNPs) outside the *DRD4* locus have not been evaluated. In this study, to re-examine the possibility of recent positive selection favoring the *DRD4* 7R allele, we genotyped HapMap subjects for *DRD4* VNTR, and conducted several neutrality tests including long range haplotype test and iHS test based on the extended haplotype homozygosity. Our results indicated that LD of the *DRD4* 7R allele was not extended compared to SNP alleles with the similar frequency. Thus, we conclude that the *DRD4* 7R allele has not been subjected to strong recent positive selection.

## Introduction

The human dopamine receptor D4 (*DRD4*; MIM 126452) gene, located on chromosome 11p15.5, contains a 48-bp variable number of tandem repeat (VNTR) in exon 3, encoding the third intracellular loop of this dopamine receptor. Ten VNTR alleles with two (2R) to eleven (11R) repeats have been identified so far. Of these, the *DRD4* 4R and 7R alleles are commonly observed in human populations [1], while the *DRD4* 7R allele is very rare in East Asians [2]. Of particular interest, the receptor encoded by the *DRD4* 7R allele has functional properties different from those by the *DRD4* 2R and 4R alleles. The DRD4 7R protein, compared with DRD4 2R and DRD4 4R, has different binding affinity to clozapine and spiperone [1], and shows a blunted intracellular response to dopamine [3]. In addition, the repeat sequence of the *DRD4* 7R allele suppresses the expression level of *DRD4* compared to the *DRD4* 2R and 4R alleles [4]. Furthermore, the *DRD4* 7R allele has been reported to be associated with behavioral and psychiatric phenotypes such as novelty seeking [5], [6] and attention-deficit hyperactivity disorder [7], [8], even though there are conflicting results [9], [10], [11], [12], [13].

The analysis of the DNA sequences of the *DRD4* VNTR alleles and their nucleotide variations suggested that the *DRD4* 7R allele originated from multiple mutational events [14], [15]. Thus, the *DRD4* 7R allele observed in human populations is considered to have a single origin. Besides the increased frequency of the *DRD4* 7R allele, it has been reported that the *DRD4* 7R allele, unlike 4R, is in strong linkage disequilibrium (LD) with the other *DRD4* polymorphisms spanning 6.3 kb [16]. Based on these unique features of the *DRD4* 7R allele compared to the most common *DRD4* 4R allele, it has been hypothesized that the *DRD4* 7R allele has been subjected to positive selection [14], [16].

The extended LD has been regarded as a signature of recent positive selection [17], [18], [19]. However, the genomic region of 6.3 kb previously studied [16] seems to be too narrow to evaluate the possibility of recent positive selection favouring the *DRD4* 7R. After the original work [16], the international HapMap project [20], [21] provided us a map of several million well-defined single nucleotide polymorphisms (SNPs) in the human genome, and the information about the SNP genotypes of subjects from African, European, and Asian populations are freely available in the public domain. The use of the HapMap data allows us to easily evaluate the degrees of LD of the *DRD4* VNTR alleles, since no further genotyping for SNPs flanking the *DRD4* locus is necessary. In this study, to re-examine whether the *DRD4* 7R allele has been the target of recent positive selection, we genotyped DNA samples from the HapMap subjects for the *DRD4* VNTR, and conducted neutrality tests including the Long-Range Haplotype (LRH) test [18] and iHS test [22], which are based on the extended haplotype homozygosity (EHH).

## Results

### Allele frequencies of the *DRD4* VNTR

We detected seven VNTR alleles with different lengths (2R, 3R, 4R, 5R, 6R, 7R, 8R) in four HapMap populations [20], [21]: YRI (Yoruba in Ibadan, Nigeria), CEU (CEPH Utah residents with ancestry from northern and western Europe), JPT (Japanese in Tokyo, Japan), and CHB (Han Chinese in Beijing, China) (Table 1). The population frequencies of the *DRD4* 7R allele were relatively high in African (YRI) and European (CEU) populations, while very low in East Asian (JPT and CHB) populations as reported in the previous study [2]. In this study, the *DRD4* VNTR alleles with the same repeat length but with the different VNTR sequences were not distinguished.

**Table 1:** Allele frequencies of the *DRD4* VNTR in HapMap populations.

	Allele									
Population	2R	3R	4R	5R	6R	7R	8R	Observed *F*	Expected *F*	P-value^a^
YRI	5	1	68	7	0	30	1	0.45	0.44	0.61
(2N = 112)	(0.04)	(0.01)	(0.61)	(0.06)	(0.00)	(0.27)	(0.01)			
CEU	10	4	78	0	1	16	1	0.53	0.44	0.77
(2N = 110)	(0.09)	(0.04)	(0.71)	(0.00)	(0.01)	(0.15)	(0.01)			
JPT	12	0	62	2	1	1	0	0.66	0.47	0.85
(2N = 78)	(0.15)	(0.00)	(0.79)	(0.03)	(0.01)	(0.01)	(0.00)			
CHB	12	1	72	2	1	0	0	0.69	0.48	0.87
(2N = 88)	(0.14)	(0.01)	(0.82)	(0.02)	(0.01)	(0.00)	(0.00)			

Note: ^a^ P-value from Ewens-Watterson test; Allele frequencies are in parentheses.

To investigate whether the observed distribution of allele frequencies at the *DRD4* VNTR is deviated from the expectation under neutrality, we performed the Ewens-Watterson homozygosity test [23], where the sum of squared allele frequencies (*F*) was calculated for the observed data, and the observed *F* value was compared with the expected ones generated by simulating samples under neutrality. The P-value was calculated as a proportion of the simulated *F* value identical or smaller than the observed *F* in the 10,000 samples simulated, which allowed us to examine both the excess and the deficiency of *F*. The observed *F* values in the HapMap populations are slightly larger than the expected ones (Table 1). However, no population showed the significant difference from the neutral expectation in two-sided test (i.e., 0.025<P-value <0.975).

### Heterozygosity around the *DRD4* locus

**Figure 1:**
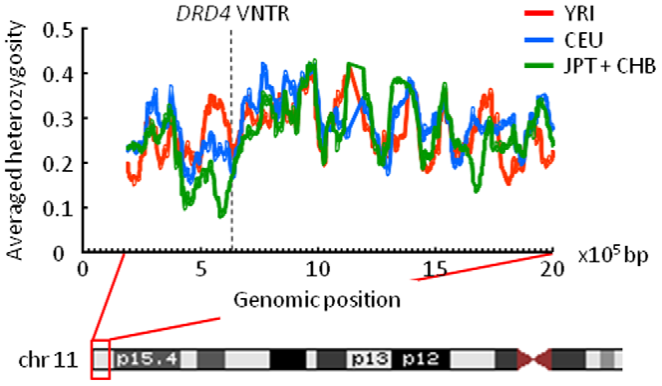
The averaged heterozygosity around *DRD4* in HapMap populations. The X axis represents the genomic position of SNP on chromosome 11. The Y axis represents the averaged heterozygosity calculated for SNPs within 25 kb on either side of the focal SNP. The allele frequency data were obtained from the HapMap database, and only SNPs that were analyzed in three HapMap populations (i.e., YRI, CEU, and JPT+CHB) were selected for the calculation. The dashed line indicates the position of the *DRD4* VNTR.

A recent positive selection alters patterns of genetic variation in the genomic region adjacent to the targeted gene. During fixation of an advantageous mutation, the variation in the surrounding region is expected to be eliminated due to the hitchhiking effect. In actual, a remarkable reduction in heterozygosity around the *ABCC11* gene, which has been subjected to recent positive selection, is observed in East Asian populations [24]. To detect signature of recent positive selection, the degree of heterozygosity around the *DRD4* locus was evaluated. Figure 1 shows the averaged heterozygosity across SNPs within 25 kb on either side of the focal SNP in a 1.8-Mb genomic region containing the *DRD4* locus in three HapMap populations (i.e., YRI, CEU, and JPT+CHB). The degree of heterozygosity at SNPs close to the *DRD4* VNTR was not lower than that of the surrounding region in any HapMap population, implying that any derived allele at *DRD4* has not rapidly reached a high frequency (e.g., >0.75). In other words, the *DRD4* locus has not experienced a strong and recent selective sweep.

### Genetic differentiation between HapMap populations

Since a local positive selection results in high genetic differentiation between two populations when one population is under selection and the other is not, the high differentiation can be regarded as an evidence of local selection operating at the locus or genomic region. Considering higher population frequencies of the *DRD4* 7R allele in YRI and CEU than JPT and CHB (Table 1), local positive selection against the 7R allele may have acted in YRI and CEU populations. We examined the degree of differentiation, measured with *F*_st_, for YRI and CEU as candidate populations. The *F*_st_ values of the *DRD4* 7R allele between YRI and JPT+CHB and between CEU and JPT+CHB were 0.15 and 0.07, respectively. We compared these values with those of SNPs on chromosome 11. The comparison revealed that 26220 and 30918 SNPs had *F*_st_ values larger than the *DRD4* 7R allele between YRI and JPT+CHB and between CEU and JPT+CHB, respectively (the empirical P-values were 0.17 and 0.20 for YRI and CEU, respectively). The analysis of *F*_st_ provided no evidence of local positive selection acting against the *DRD4* 7R allele.

### Structure of LD around the *DRD4* locus

**Figure 2:**
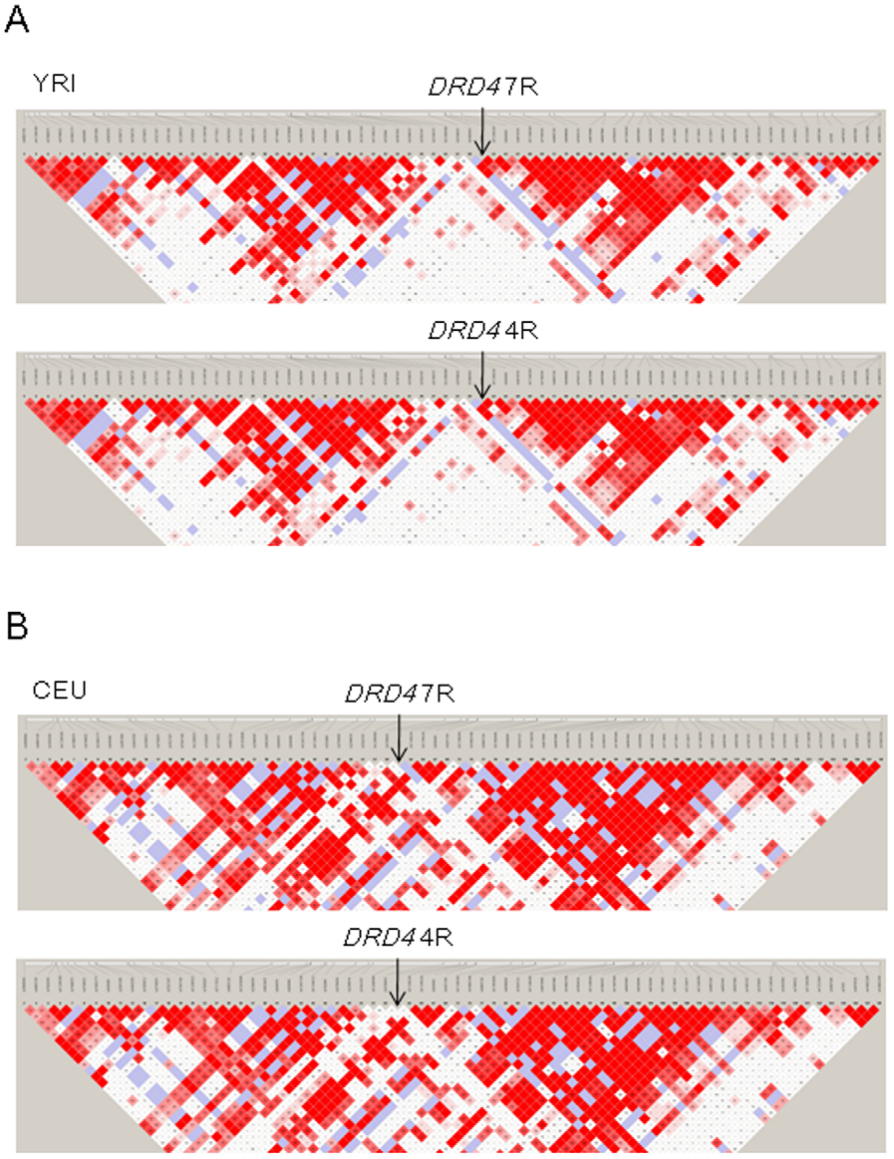
LD structure around *DRD4* VNTR. The |*D*'| values for all pairwise combinations of SNPs spanning 200 kb and *DRD4* VNTR allele (7R or 4R) are visualized. The positions of *DRD4* 7R and 4R alleles are indicated by arrows. Red squares indicate statistically significant LD (LOD >2) between the pair of loci, and darker colors of red indicate higher values of |*D*'|. Blue squares indicate |*D*'| of 1 but without statistical significance. White squares indicate |*D*'| of <1 with no statistically significant evidence of LD. (A) YRI. (B) CEU.

LD plots based on |*D*'| were visualized for YRI and CEU (Figure 2). Although the allele frequency of 7R was lower than 4R, we found that |*D*'| values from the *DRD4* 7R allele to SNPs located outside the *DRD4* locus were similar to those from the *DRD4* 4R allele.

### LRH and iHS tests for *DRD4* VNTR 7R allele

**Figure 3:**
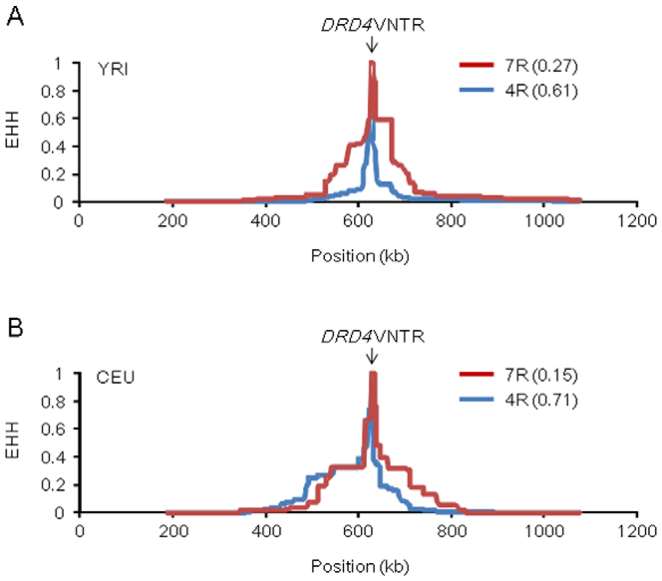
EHH values of *DRD4* 7R and 4R alleles. Red and blue lines indicate EHH of the 7R-bearing chromosomes and 4R-bearing chromosomes, respectively. The allele frequencies of *DRD4* 7R and 4R in each population are presented in parentheses. The horizontal axis represents the physical position of SNPs on the chromosome 11. The position of *DRD4* VNTR is indicated by an arrow. (A) YRI. (B) CEU.

**Figure 4:**
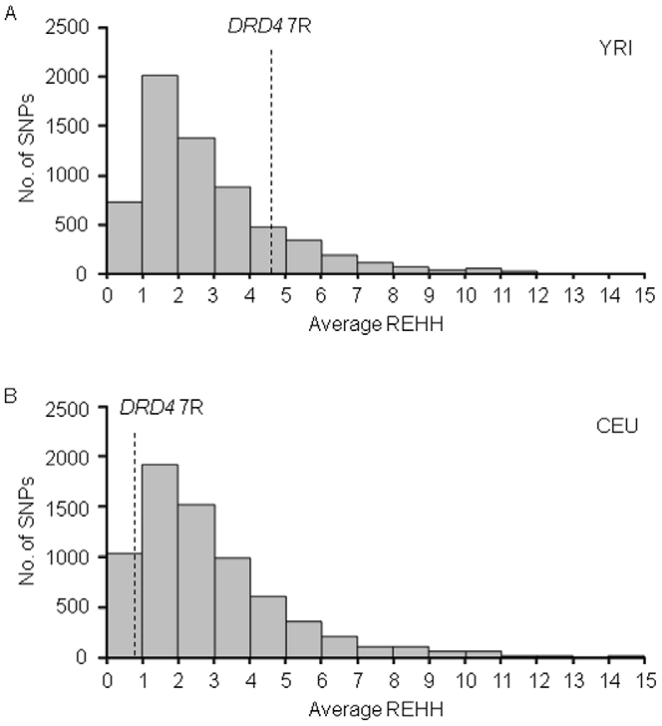
Empirical distribution of REHH of SNPs on the chromosome 11. For each SNP with an allele whose population frequency was similar to the *DRD4* 7R allele, the average of REHH values on both sides (i.e., centromere-proximal and cetromere-distal) was calculated, and the empirical distributions for SNPs on the chromosome 11 were obtained. The data of SNPs with the average of REHH more than 15 are excluded from the histogram. REHH was calculated at a distance of 0.25 centimorgans (cM) from the core SNP. REHH value of *DRD4* 7R is indicated by dashed line. (A) YRI. (B) CEU.

We next calculated the EHH values [18] of the *DRD4* 7R and 4R alleles in YRI and CEU populations (Figure 3). The 7R-bearing chromosomes have longer LD than 4R-bearing chromosomes in YRI, whereas such a tendency was not found in CEU, regardless of much lower frequency of the *DRD4* 7R allele compared to the *DRD4* 4R allele. To examine whether the LD from the *DRD4* 7R allele has been extended by recent positive selection, we used the LRH test [18] based on the relative EHH (REHH) value, in which the other allele (i.e., reference allele) at the same locus serves as an internal control to normalize recombination rate variation [25]. In this study the REHH value for the focal *DRD4* VNTR allele was calculated at a distance of 0.25 centimorgans (cM) from the *DRD4* VNTR. The EHH value of the 7R-bearing chromosomes in YRI was 4.58 times greater than the EHH of the reference chromosomes on the centromere-distal side and 4.58 times greater on the centromere-proximal side. Thus, the average of REHH values on both sides was 4.58 ( =  [4.58 + 4.58]/2). In CEU, the REHH values of the 7R-bearing chromosomes were 1.55 and 0 on the centromere-distal and centromere-proximal sides, respectively (i.e., the average REHH was 0.78). To calculate the empirical P-values of the *DRD4* 7R allele, we obtained the empirical distributions of the REHH values for SNPs whose allele frequencies were similar to the *DRD4* 7R alleles in YRI and CEU populations (Figure 4). In both populations, the observed REHH values of the 7R-bearing chromosomes were not significantly large compared to the other SNPs on the chromosome 11 (i.e., empirical P-values were 0.17 for YRI and 0.90 for CEU, respectively).

**Figure 5:**
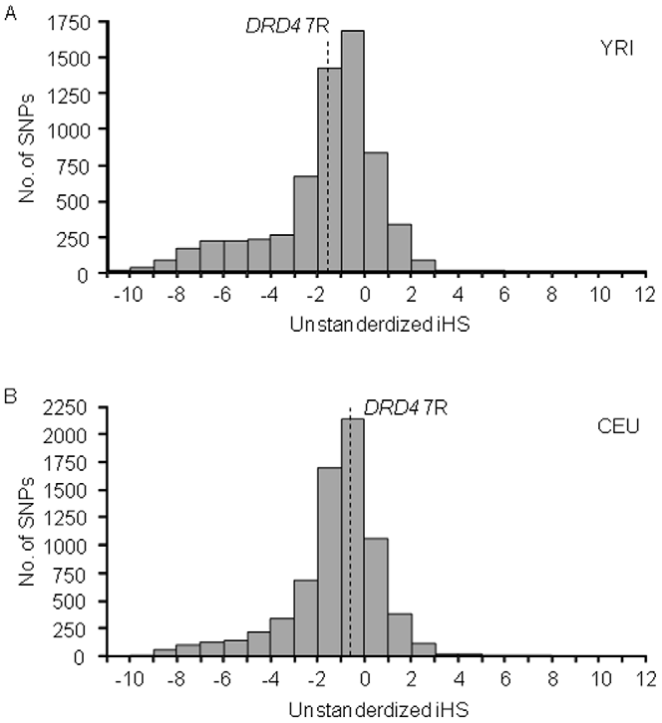
Empirical distribution of unstandardized iHS values of SNPs on the chromosome 11. For each SNP with an allele whose population frequency was similar to the *DRD4* 7R allele, the unstandardized iHS was calculated and the empirical distributions for SNPs on the chromosome 11 were obtained. The iHH value (i.e., area under the EHH curve) was calculated until EHH reaches 0.05. Unstandardized iHS value of *DRD4* 7R is indicated by dashed line. (A) YRI. (B) CEU.

We further performed the iHS test [22]. In the iHS test, unstandardized iHS value was computed for SNPs with minor allele frequency similar to the frequency of the *DRD4* 7R allele. Large negative unstandardized iHS values indicate that minor-allele-bearing chromosomes have much longer LD than major-allele-bearing chromosomes. The unstandardized iHS values of the 7R-bearing chromosomes in YRI and in CEU were -1.23 and -0.65, respectively. These values were not large negative when compared with the unstandardized iHS values for SNPs on the chromosome 11 (Figure 5). The empirical P-values for the *DRD4* 7R allele were 0.46 for YRI and 0.60 for CEU, respectively.

### Power simulation

Neither LRH test nor iHS test provided any evidence of longer LD of the 7R-bearing chromosomes. However, selection intensity for the *DRD4* 7R allele might be too low to be detected. We assessed the power of the LRH test and iHS test by using a computer simulation. Using SelSim program [26], 120 chromosomes bearing 101 SNPs evenly distributed in the region with the size of 1 centimorgan (cM) were simulated, where a derived allele at the 51th SNP was assumed to be subjected to positive selection. The final population frequency of the selected allele was set to be either 0.15 or 0.3, since the present frequencies of the *DRD4* 7R allele in CEU and YRI are approximately 0.15 and 0.3, respectively.

**Figure 6:**
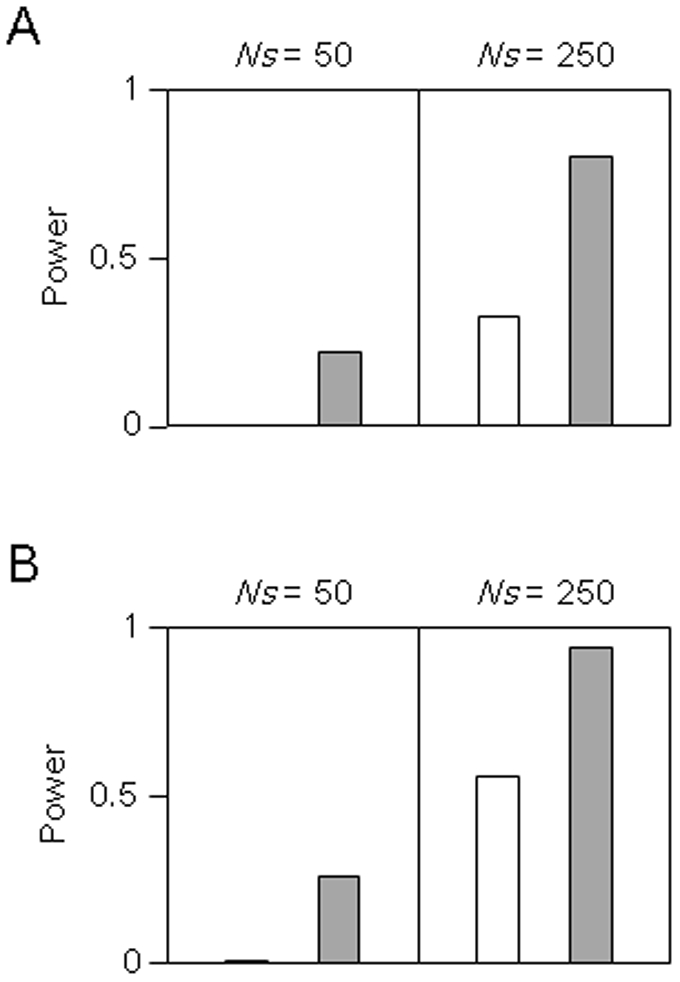
Power of LRH and iHS tests. White and shaded bars indicate the LRT and iHS tests, respectively. The final population frequencies of the selected allele are (A) 0.15 and (B) 0.3.

The results demonstrated that the power of the LRH test [18] was always smaller than that of the iHS test [22] in the present parameter settings (Figure 6). Here, *Ns* of 50 corresponds to *s* of 0.005 in a population with the population size of 10,000. Although our simulation indicated that the LRH test reveals very low statistical power for a selected allele with small selection coefficient, the LRH test successfully detected a significant evidence of positive selection acting at the *G6PD* gene [18]. Since the selection coefficient of the *G6PD* allele conferring resistance to malaria has been estimated to be more than 0.1 [27], we may say that the LRH test has enough power to detect such a strong recent selection.

The iHS test achieved high statistical power (>0.8) for *Ns* (*N* is diploid population size and *s* is selection coefficient) of 250 when the final population frequencies of the selected allele were 0.15 and 0.3, but showed low power (< 0.3) for *Ns* of 50. In addition, we should note that both LRH and iHS tests are expected to attain lower power for actual populations such as YRI and CEU than for the model assumed in our simulation because the variance of test statistic for neutral SNPs is larger in the former due to their complicated population history. Thus, statistical power of the present neutrality tests, LRH and iHS, for the HapMap subjects is too low if selection intensity has not been very high.

## Discussion

No significant extended LD from the *DRD4* 7R allele to SNPs outside the *DRD4* locus was detected in the present study. Our results seem to be inconsistent with the previous observation that the nucleotide diversity of the *DRD4* haplotype bearing polymorphisms spanning 6.3 kb is more reduced in the *DRD4* 7R allele than the most common *DRD4* 4R allele [16]. However, it is not surprising that the diversity of the *DRD4* 7R allele is lower than that of the *DRD4* 4R allele if the *DRD4* 7R allele is derived from the *DRD4* 4R allele [14].

A previous study also showed that non-synonymous substitutions have occurred more frequently than synonymous ones in the human *DRD4* VNTR [14]. This implies that diversifying selection has operated against *DRD4* VNTR especially in human lineage, though the length polymorphism at *DRD4* VNTR has been commonly found even in nonhuman primate species [28]. However, it is unlikely that the observed bias towards nonsynonymous amino-acid changes has been achieved only by positive selection favoring the *DRD4* 7R allele because the bias can be found not only in VNTR motifs of the *DRD4* 7R allele but also in those of the other alleles [14].

For selectively neutral alleles with the similar population frequencies, their age is thought to be inversely proportional to the REHH value. It is not feasible to estimate the age of the *DRD4* 7R allele based only on the present *DRD4* VNTR allele frequency data. However, the observed REHH and unstandardized iHS values for YRI and CEU (Figures 4 and 5) indicate that the age of the *DRD4* 7R allele is not extraordinarily young compared to SNP alleles with the similar frequencies. Hattori et al. [15] pointed out the possibility that five distinct *DRD4* VNTR alleles had been derived from the *DRD4* 7R allele based on the sequencing analysis of the VNTR region. If this prediction is true, the original *DRD4* 7R allele is considered to have appeared long time ago. These observations lead us to conclude that the *DRD4* 7R allele has not been subjected to strong recent positive selection and the increased population frequencies of the *DRD4* 7R allele in African and European populations would have been caused by random genetic drift, though the possibility that diversifying selection has acted against the *DRD4* VNTR in primate species including humans for a long evolutionary time cannot be excluded.

## Materials and Methods

### Samples

The genomic DNA samples used by the International HapMap Project were obtained from the Coriell Cell Repository: 56 subjects from YRI (Yoruba in Ibadan, Nigeria), 55 CEU (CEPH Utah residents with ancestry from northern and western Europe), 39 JPT (Japanese in Tokyo, Japan), and 44 CHB (Han Chinese in Beijing, China).

### Genotyping

For genotyping the VNTR in exon 3 of the *DRD4* gene, PCR was performed using the following pair of primers previously reported [29]: upstream 5′-ACTACGTGGTCTACTCGTCCGTGT-3′ and downstream 5′-TCAGGACAGGAACCCACCGA-3′. PCR was performed with an initial denaturation at 95°C for 5 min, followed by 40 cycles of denaturation at 95°C for 30 s, annealing at 60°C for 45 s, and extension at 72°C for 30 s, and a final extension at 72°C for 7 min using a thermal cycler (GeneAmp PCR system 9700; Perkin-Elmer Applied Biosystems). The PCR-amplified DNA fragments were analyzed using a microfluidics-based platform (Agilent 2100 bioanalyzer; Agilent Technologies) for accurate sizing with the Agilent 2100 Bioanalyzer DNA 1000 kit (Agilent Technologies).

### Statistical analyses

Using Arlequin version 3.5 [30], the Ewens-Watterson test [23] based on Ewens sampling theory of neutral alleles [31] was performed to test whether the observed distribution of allele frequencies at the *DRD4* VNTR is deviated from the expectation under neutrality.

The allele frequency data of SNPs on the chromosome 11, analyzed in all the HapMap populations, were obtained from the HapMap database (http://hapmap.ncbi.nlm.nih.gov/index.html.en) and used for the calculation of the heterozygosity. The heterozygosity was calculated for each SNP and then averaged across SNPs within 25 kb on either side of the focal SNP in three HapMap populations (i.e., YRI, CEU, and JPT+CHB).

A pairwise *F*_st_ value for each SNP on the chromosome 11 was calculated between HapMap populations based on the following formula [32]: *F*_st_  =  (*H*_A+B_ – [*H*_A_+*H*_B_]/2)/*H*_A+B_, where *H*_A_  = 2*p*_A_(1-*p*_A_), *H*_B_  = 2*p*_B_(1-*p*_B_), and  = 2([*p*_A_+*p*_B_]/2)(1-[*p*_A_+*p*_B_]/2). Here, *p*_A_ and *p*_B_ represent the population frequencies of one of alleles for each SNP in populations A and B, respectively. For the *F*_st_ value of the *DRD4* 7R allele, *p*_A_ and *p*_B_ are the population frequencies of the *DRD4* 7R allele in populations A and B.

To evaluate the structure of LD around the *DRD4* VNTR, the SNP genotype data of HapMap subjects were obtained from the HapMap database, and the absolute *D*' values, |*D*'|, for all pairwise combinations of SNPs with minor allele frequency of more than 0.1 and *DRD4* VNTR allele were estimated and visualized using Haploview software [33]. Here, SNPs spanning 200 kb were analyzed. As Haploview program is unable to analyze multi-allelic locus such as VNTR, only one of VNTR alleles was focused and the other VNTR alleles were regarded as the same ones. For example, the *DRD4* 7R allele and the other VNTR alleles were designated as “A” and “G”, respectively for Haploview analysis.

The phased haplotypes or diplotype in an individual for the calculation of EHH [18] were estimated by using fastPHASE program version 1.2.0 [34]. Like Haploview analysis, in fastPHASE analysis, only one of VNTR alleles was focused and the other VNTR alleles were regarded as the same ones. To conduct the LRH test [18], the empirical distributions of the REHH of SNPs on the chromosome 11 were obtained for YRI and CEU. Only SNPs with similar allele frequency of the *DRD4* 7R allele were considered. The phased haplotype data were retrieved from the HapMap database. The REHH value was defined as the ratio of the EHH on the tested allele compared with the EHH of the reference allele (i.e., the other allele) at a distance of 0.25 cM from the core SNP. When the EHH of the reference allele was 0, the REHH value was excluded from the empirical distribution.

To perform the iHS test [22], in plots of EHH versus distance, the area under the EHH curve was calculated until EHH reaches 0.05. This integrated EHH (iHH) (summed over both directions away from the core SNP) was computed for each allele of SNPs with minor allele frequency similar to the frequency of the *DRD4* 7R allele, and was denoted iHH_major_ for major allele or iHH_minor_ for minor allele. In this study, test static iHS was defined as follows: 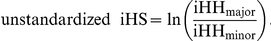



Large negative values therefore indicate long haplotypes carrying the minor allele. Unlike the original study [22], we did not consider the ancestral status of each SNP allele because the ancestral state of each *DRD4* VNTR allele is unknown.

### Computer simulation for power calculation

Using SelSim program [26] based on the coalescent theory, 120 chromosomes bearing 101 SNPs evenly distributed in the region with the size of 1 cM were simulated assuming a population with constant size, *N*, of 5000 individuals. In the simulation, a derived allele at the 51th SNP was positively selected with selection coefficient of *s*, where the dominance parameter was set to 0.5 (i.e., genic selection). It should be noted that the product of *N* and *s* (i.e., *Ns*) is important rather than each parameter in this coalescent simulation. For 120 chromosomes, the EHH values of the derived and ancestral alleles at the 51th SNP for both directions were computed. REHH value at a distance of 0.25 cM from the selected SNP and iHH value were calculated for LRT test [18] and iHS test [22], respectively. We performed 200 simulation runs for deterministic models with *s* of 0.01 (i.e., *Ns* of 50) and 0.05 (i.e., *Ns* of 250). In addition, 1000 runs for neutral model (*s* = 0) were performed to have the null distributions of the average REHH and unstandardized iHS values. In the calculation of the statistical power, “positive selection” was regarded to be successfully detected in a simulation run when the test statistic (average REHH in LRH test or unstandardized iHS in iHS test) in selection model exceeded the 95th percentile of those observed in neutral model. The proportion of the detection in 200 runs was defined as the statistical power.
